# Knowledge-graph-based explainable AI: A systematic review

**DOI:** 10.1177/01655515221112844

**Published:** 2022-09-24

**Authors:** Enayat Rajabi, Kobra Etminani

**Affiliations:** Shannon School of Business, Cape Breton University, Canada; Center for Applied Intelligent Systems Research (CAISR), Halmstad University, Sweden

**Keywords:** Knowledge graph, artificial intelligence, systematic review, explainable AI

## Abstract

In recent years, knowledge graphs (KGs) have been widely applied in various domains for different purposes. The semantic model of KGs can represent knowledge through a hierarchical structure based on classes of entities, their properties, and their relationships. The construction of large KGs can enable the integration of heterogeneous information sources and help Artificial Intelligence (AI) systems be more explainable and interpretable. This systematic review examines a selection of recent publications to understand how KGs are currently being used in eXplainable AI systems. To achieve this goal, we design a framework and divide the use of KGs into four categories: extracting features, extracting relationships, constructing KGs, and KG reasoning. We also identify where KGs are mostly used in eXplainable AI systems (pre-model, in-model, and post-model) according to the aforementioned categories. Based on our analysis, KGs have been mainly used in pre-model XAI for feature and relation extraction. They were also utilised for inference and reasoning in post-model XAI. We found several studies that leveraged KGs to explain the XAI models in the healthcare domain.

## 1. Introduction

Most Artificial Intelligence (AI) systems operate using very complex criteria, which makes it difficult for humans to understand and interpret the rationale behind the decisions made by a given model [1]. The proliferation of AI systems has increased the need for models that can generate ‘explanations’, as such explanations can provide users with an overview of the system, which in turn can educate them on how it functions and assists them in future explorations [2]. Although the terms, ‘explainability’ and ‘interpretability’, are used interchangeably in the field of AI, there are discrepancies in how they are defined in the literature. ‘Explainability’ typically refers to any technique that helps the user of a machine learning model understand the model’s behaviour and performance [3]. Explainability makes an AI system more understandable, transparent, interpretable, auditable, and responsible, while also reducing risks. Recently, researchers have developed several eXplainable AI (XAI) systems capable of generating explainable models or predictions, thus enabling users to better understand the AI system and its decisions [4]. Most XAI applications can explain what has been done previously, what is being done currently, and what will be done in the future.

On the other hand, Semantic Web technologies are used to structure data, extract the features and relationships in a system, and explain a model through reasoning using common vocabularies and ontologies [5]. In a semantic model, ontologies can be applied to represent knowledge hierarchically via classes of entities (concepts), their properties (roles), and their relationships [6]. One of the more attractive concepts associated with the Semantic Web are knowledge graphs (KG), which are very large semantic nets that integrate heterogeneous information sources to represent knowledge about certain domains of discourse [7]. In a KG, knowledge is represented in a graph to allow a machine to provide meaningful answers to queries ( ‘questions’) via reasoning and inference [8,9]. The combined use of KGs and machine learning models can make AI systems more transparent and interpretable, as machine learning models are capable of extracting relations, features, and entities, as well as inferring new concepts. KGs can be used to answer questions, understand images, and retrieve information, which are all relevant aspects of many types of research. Recently, the integration of AI systems into coherent and comprehensive KGs has emerged as an open challenge [10]. In this article, we conduct a systematic review of how and where KGs are being used in existing XAI systems. To this end, we review scholarly publications that examine several dimensions of KG-based XAI and categorise them based on how/where they leverage KGs in XAI. Researchers and experts can also use this review to identify and highlight the areas where KGs have been used most and least often. This article makes two major contributions:

- We conduct a systematic review to identify recently published studies that use KGs for explainability purposes.
- We present a framework to identify how KGs have been used in various XAI models.

In the next section of this article, we explain the survey methodology, research questions, and the eligibility criteria, while the following section (conceptualisation section) provides an overview of the concepts and dimensions considered in the survey. Next, the literature section provides quantitative and qualitative analysis of the reviewed articles and presents the proposed framework. Finally, we conclude our article by deriving insights from the gap analysis.

## 2. Survey methodology

**Figure 1.**
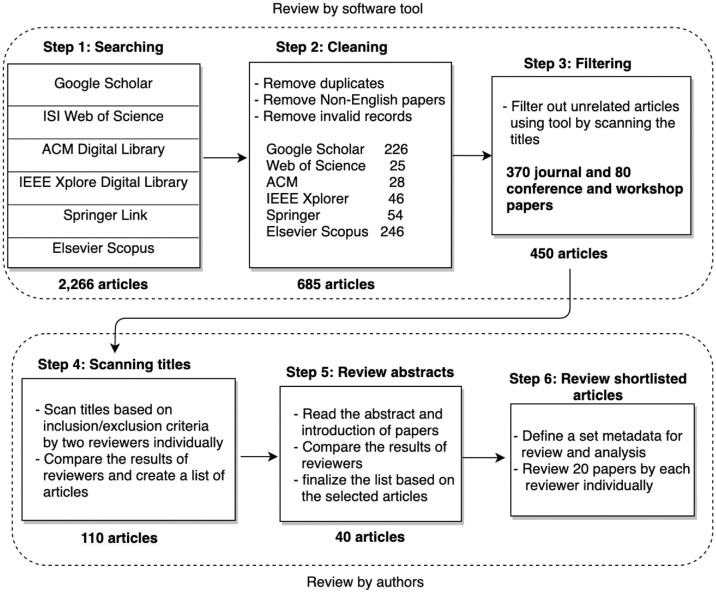
The systematic review methodology.

This systematic review presented herein was conducted by the authors of this article following the systematic review procedures described in Kitchenham (11). Specifically, we sought to tackle the following problems: (a) we sought to summarise and compare existing KG-based explainable AI approaches, and (b) we sought to determine the contributions of various approaches in terms of how and where they used KGs for the purposes of explainability. An overview of our search methodology, including the number of articles retrieved in each step, is shown in Figure 1 and described in detail below. Furthermore, a forward and backward search (2) was also conducted to complement the list of relevant research articles.

## 3. Related surveys

We studied related surveys and literature reviews to justify the current systematic review. The importance of the role of KGs was highlighted in Lecue [5], wherein the authors reviewed articles focusing on XAI in relation to both machine learning and other AI-related research topics. In this review, the authors discussed the main challenges of XAI, along with various existing approaches and their limitations and opportunities for Semantic Web technologies.

Elsewhere, Seeliger et al. [12] conducted a literature review aimed at connecting machine learning models and Semantic Web technologies. In this review, the authors considered four general aspects of the Semantic Web technologies: ontology, KG, taxonomy glossary, and lexicon. Their results highlighted how Semantic Web technologies provide semantically interpretable tools that enable reasoning to be performed on knowledge bases and facilitate explanations in machine learning models, including artificial neural networks.

Wohlin [2] conducted a review of the literature related to explainable artificial intelligence systems, with a focus on knowledge-enabled systems, including expert systems, cognitive assistants, semantic applications, and machine learning domains. In this review, Wohlin proposed new definitions for explainable knowledge-enabled systems based on his findings. According to this study, the two main considerations in developing explainable knowledge-enabled systems are enabling knowledge utilisation to provide intuition for the functioning of unintelligible models and building a vocabulary to explain the algorithms’ conclusions/inputs/workings. In addition, Wohlin also observed that prior knowledge of the requirements of explanations in the form of taxonomies can serve as checks for future explainable models.

In their survey article, Burkart and Huber [13] provided a detailed introduction to explainable supervised machine learning models that focused on defining and classifying the various approaches in the field. In doing so, they discussed the reasons for explainability in machine learning models and highlighted the role of ontologies (as one explanation method) in generating explanations in different supervised machine learning models. Their review, which examined several articles, showed that explanations based on semantics and knowledge in the ontologies and KGs can be used to provide context-specific information that can enrich data for machine learning. The explanations are generated using a KG, and the contextual information from the machine learning is used to identify representative semantic relations.

Despite the interesting findings of these surveys and studies, the question of how and where KGs are typically used in machine learning models remains unanswered. Thus, we will explore this research gap in the present article.

### 3.1. Research questions

This review examines the existing methodologies for leveraging KGs in explainable AI systems, including machine learning algorithms. To achieve this goal, we seek to answer the following research question: ‘Can KGs be used to achieve explainability and interpretability in AI-based systems?’ This general question is divided into the following sub-questions:

- What kinds of XAI and machine learning models have used KGs for explainability?
- Where in the AI process were KGs utilised in these XAI models?
- How did these XAI models use KGs to achieve explainability?

### 3.2. Eligibility criteria

We developed the following inclusion and exclusion criteria to help determine which of the identified scholarly articles to include in this study.

Inclusion criteria:

- Study must have been published in English between 2018 and 2021.
- Study must focus on leveraging KGs for explainability/interpretability in AI-based systems.

Exclusion criteria:

- Article uses different algorithms for KG completion or link prediction.
- Study focuses on recommendation systems.
- Review, prototype, report, general idea, or proposal articles, and studies that do not propose any methodology, approach, or framework for explainability.

### 3.3. Search strategy

Based on the research questions and the eligibility criteria, the authors determined that the following terms were most appropriate for this systematic review:

*(explainable) AND (artificial intelligence OR AI) AND (knowledge graph)*

The authors categorised a article as a candidate for inclusion if its title or abstract contained the abovementioned keywords . To aid in this search, we built a software tool to detect the selected keywords in the titles and abstracts of articles, and to retrieve relevant articles from different research databases. The tool was written in Python programming language to enable access to the metadata of publications using the API provided by the academic databases. The tool used the keyword search in the following list of search engines, digital libraries, journals, and conferences and their respective workshops: Google Scholar, ISI Web of Science, ACM Digital Library, IEEE Xplore Digital Library, Springer Link, and Elsevier Scopus. The search tool found 2,266 articles from these databases; of these articles, 1,581 were either duplicates, books, not written in English, or invalid records. We again used Python to perform the cleaning and preparation steps, and we added more metadata, such as citations, to the dataset using Google Scholar API ^1^.

We also used Python to filter out any articles that did not meet the defined eligibility (e.g. articles with ‘recommendation system(s)’ in their title, books, and conference proceedings, etc.). In total, the tool identified 450 articles eligible for review. The two authors then independently scanned the titles of the identified articles to assess their eligibility and inclusion/exclusion criteria. The authors compared their results and agreed on 110 articles to be analysed further in the next step.

Search strategies usually follow an iterative process. Given that it is not possible to determine a article’s relevance by simply reviewing its title, the authors agreed to review the abstracts of the remaining 110 articles separately to avoid bias and to ensure the complete coverage of all related articles. Ultimately, 40 articles were short-listed by both authors as being most relevant based on the defined criteria. Disagreements between the authors after screening the titles and reading the abstracts were resolved either by achieving mutual consensus or by creating a list of articles to go under a more detailed review. The article-selection process is illustrated in Figure 1. We also identified five survey articles during the selection process and reviewed them separately. These articles were also included in this study.

With respect to the review strategy, each author reviewed 20 articles based on the dimensions discussed in the three research sub-questions. Each of these dimensions will be discussed in the following section.

## 4. Conceptualisation

In this section, we discuss the dimensions that were defined in the research questions. These dimensions were identified after examining the abstracts, introductions, and the conclusions of the selected articles. Each dimension is analysed individually this section, and classified quantitatively and qualitatively in the Literature section.

### 4.1. How to use KGs

KGs are used to describe entities and their relationships in the real world. They can be utilised in general areas or domain-specific cases, and they can also be used to build intelligent search engines, question-answering systems, social networks, and machine translation systems in a range of different domains (e.g. education, healthcare, etc.). The dimension of ‘how to use a KG’ relates to the following question: ‘how does a study leverage a KG for the purpose of explainability?’ To answer this question, we catalogued the ways in which KGs are used in the reviewed articles and divided these uses into the following four categories:

- KG construction: A KG can be composed of different concepts (e.g. drugs, symptoms), synonyms for concepts, hierarchical and associative relations between concepts, and mappings to the other concepts in external terminologies used in different knowledge-based systems. Thus, many data publishers and providers construct KGs to organise their data, annotate and present different types of information in a meaningful way, and add semantic labels or tags to a set of information.
- Feature extraction: Many knowledge-embedding methods use KGs to extract features in various domains. For example, KGs are used in text analytics to identify entities and features in a text, which are then used to create semantically structured summaries in order to enrich search results. KGs are also utilised to provide links to related entities, improve search engines’ capabilities, and enhance the user’s search experience.
- Relations extraction: A KG can be used to represent and integrate knowledge encoded in various standards. This approach creates semantic relationships between different entities, and it allows interoperability conflicts among standardisation frameworks and standards to be resolved. Furthermore, KGs can be applied in integrated search engines to present a summary of relevant information about search queries and a list of related topics.
- KG Reasoning: KGs can be used in reasoning to accurately predict truth or expressions based on existing information or facts. New facts and conclusions can be inferred from the existing entities, concepts, and relationships in a KG; hence, this is one of the dimensions that should be considered in this systematic review.

### 4.2. Where to use KGs

KG explainability can be leveraged at different stages in the AI development pipeline [4]. KG explainability is usually performed before (pre-modelling explainability), during (explainable modelling), or after (post-modelling explainability) the AI modelling stage [14].

- Pre-modelling: A pre-modelling explainability method functions independently of the model and usually employs a KG prior to model selection, as it is only applicable to the data itself. Pre-modelling explainability methods can fall into different categories, such as constructing KGs from a dataset or standardising a dataset with KGs.
- In-modelling: In-modelling explainability focuses on the model’s inner workings (e.g. its mathematical aspects) and uses KGs to generate explanations during the training phase of model creation.
- Post-modelling: Post-modelling explainability techniques usually describe the application of a KG after the training of a model [15]. These techniques improve the explainability of AI after the model has been built, and they use KGs to provide insights into what the trained model has learned, without changing the underlying model.

### 4.3. Which machine learning models use KGs

As a branch of artificial intelligence, ‘Machine learning is the study of computer algorithms that allow computer programs to improve through experience automatically’ [16]. Machine learning employs a variety of statistical, probabilistic, and optimization techniques that allow computers to ‘learn’ from past examples and detect hard-to-discern patterns from large, noisy, or complex data sets [17]. In this survey, we identified the specific machine learning model by reading the methodology section of each article. If a study used a specific and customised version of a machine learning model (e.g. a customised version of a convolutional neural network), we considered the general model type (e.g. convolutional neural network) for our study.

## 5. Literature review

In the following subsections, we analyse the reviewed articles from a quantitative and qualitative perspective.

### 5.1. Quantitative analysis of short-listed articles

**Figure 2.**
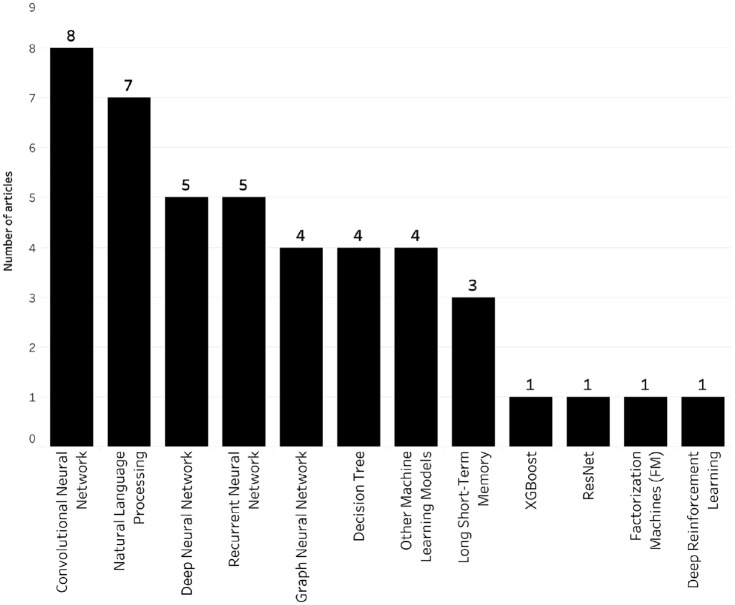
Artificial intelligence aproaches.

Of the 40 short-listed articles, 24 leveraged KGs in the pre-model phase, nine used KGs in the in-model phase, and eight used KGs in the post-model phase of XAI system development. It should be noted that one article used KG in both the pre-model and in-model phases. Most of the machine learning models used in the analysed studies were based on neural network models. As shown in Figure 2, the largest proportion of studies (23%) used convolutional neural network models in their XAI system, followed by natural language processing (20%) and deep neural networks (14.3%). In terms of KG utilisation, 16 studies included the construction of a KG for explainability, while 30 articles explained the utilised model by feature or relation extraction, and 10 used KGs for inference and reasoning. It should be noted that some articles used KGs for two or more applications.

**Figure 3.**
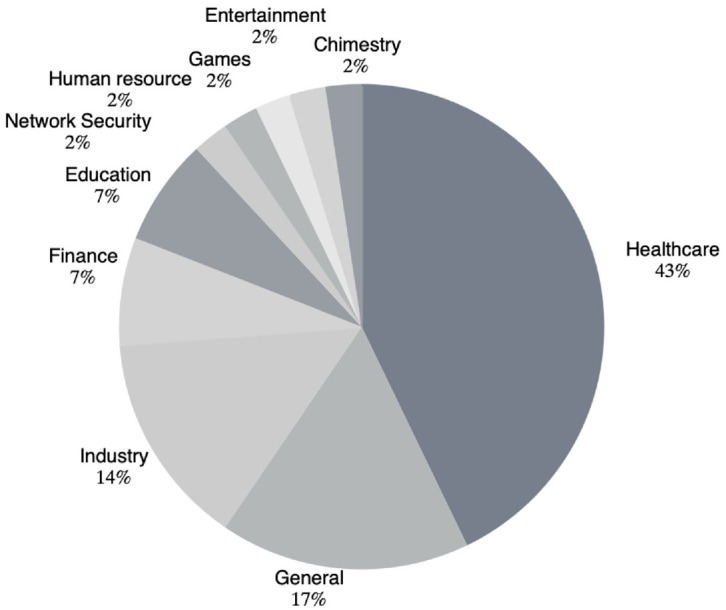
The areas in which KG was used.

As can be seen in Figure 3, KGs were predominantly leveraged for explainability in the healthcare domain (43%). For example, KGs were incorporated into neural-network-based models to extract features for disease or drug classification [9,18] or drug reaction problems [19,20]. KGs have also been utilised in decision-support systems in healthcare to assist with medical reimbursement decisions, image report generation, and the collection of statistics related to morbidity and mortality [21,22]. Other important areas in which KGs have been utilised for different purposes include education (7%) and finance (7%). In the education domain, for example, KGs have been used in AI-based education systems to integrate the teaching experience and domain knowledge of discipline experts to enhance explainable and robust machine intelligence [23]. Reasoning in KGs has also been gaining more attention in the finance domain. For instance, KGs have been applied in different studies to perform reasoning and find missing information in tax refunds [24], as well as to extract structured events from financial news and to provide external knowledge that can be used to embed events and forecast stock trends [25].

In several studies, the authors created graphs or complex networks from text based on similarities between paragraphs in a text or different documents [26,27]. KGs have also been applied in Natural Language Processing (NLP) tasks such as answering questions or classifying documents [28], as well as mining explanation rules from text to identify consistent semantic preferences [29].

Researchers have applied KGs to help their models learn representations from text in order to provide explanations that will enable decision-making based on partial observations [30].

In terms of working with images, KGs have been used in conjunction with deep learning to help protect image privacy and to infer the privacy risk associated with an image [31]. In addition, KGs have been used to predict the images of new classes (i.e. unseen classes) that were not included in the training set by transferring features learned from the training classes [32,33].

### 5.2. Qualitative analysis of short-listed articles

KGs have been widely used in a variety of machine learning models. In this study, we design a framework that: a) provides greater insight into how KGs are applied to enhance the explainability of machine learning models, and b) assists in answering our research questions. We considered each research question as a distinct dimension, and we categorised the machine learning models based on KG usability and XAI type (Table 1).

**Table 1.** The designed framework based on explainability type, the use of KG, and machine learning model.

XAI Type	KGC	FE	RE	RS
Pre-model	RNN (34) DNN (35)	CNN (32,36)		
	NLP (24,37,38) CNN (25,39)	LSTM (40) DNN (41)	GNN (42) LSTM (40,43)	
	GNN [31] DRL [30]	NLP (37,44)	NLP (38,45)	
	NB [19] Clustering [23]	CNN (46) DNN (47)	RL (48) Clustering (23)	
	LSTM (40,43)	NB (19) DT (9,20)		
In-model	RNN (49)		DT (50) RNN (28)	NLP (24,45)
	NLP (51)	DNN [33]	GNN (52) FM (53)	DT (37)
	GNN [21]		ResNet (54)	DRL [30]
Post-model		XGBoost (ensemble) [55]		DNN (47)
		CNN, RNN [22]	NN (56)	LSTM [29]
		GNN (42) DT (57)	GRU [18]	CNN (29,36,39)
				DNN (35,58)

According to our analysis, KGs were mainly applied in pre-modelling compared with other XAI types. In pre-model XAI, the majority of studies leveraged different neural-network-based models (e.g. CNN (21,25,32,39), GNN (31), RNN (34), and LSTM (40,43)) to extract features from KGs for different purposes, including the extraction of entities from text. In another study (32), a KG was used to transfer features in a zero-shot learning model and to generate explanations of unseen classes in an image-classification problem. In the healthcare domain, one study (20) used a KG to identify human-readable bio-molecular features in order to enable automatically reproducing expert classifications distinguishing drugs that do or do not cause a given type of adverse reaction.

Constructing KGs from text or medical codes was another popular pre-modelling application. To take an example, in Huo et al. (43), the authors constructed a multi-spatial knowledge graph to demonstrate the effectiveness of KGs in context embedding and their superiority over complex feature engineering. The authors of Zhao et al. (40) also created a KG from Electronic Medical Records (EMR) and used the information stored within in the diagnosis process of a Bi-LSTM model to further enhance its performance. Elsewhere, researchers (37) constructed a disease-related KG to extract knowledge from the real-world clinical and pathological data of thousands of patients diagnosed by hundreds of expert doctors. To this end, the authors used a decision tree to implement categorical reasoning in the KG for deductive decision making, and they added a Semantic Engine (Reasoning Knowledge Network) to help health service providers make accurate, informed decisions on balancing reactive care through explainable AI. In another example, Wang et al. (19), a KG was created based on MEDLINE articles^2^ using an NLP tool (Apache cTAKE) to extract entities from free medical texts in order to identify adverse drug reactions.

In terms of relation extraction, Deep Learning (38,45), LSTM (40,43), and GNN (42) were the machine learning models most commonly used in pre-model AI systems. The authors of (48) incorporated background knowledge about gene function, associated pathways, known drug targets, and cancer cell type into a Relational Learning (LR) model and an Aleph inductive logic programming engine based on a breadth-first search to learn and understand the mechanisms of cancer drugs. Our review revealed no studies wherein KGs were used for inference and reasoning in the pre-model XAI system, thus indicating that reasoning is usually applied after developing machine learning models.

Other studies, such as previous works (22,42,55,57), used KGs for feature and relation extraction in post-model XAI. For example, the authors of Cui et al. (42) applied a KG to a graph neural-network model to capture the important features and relations in a set of news articles and used the relevance scores of entities to guide the embedding of the article. In another article, Li et al. (22), both convolutional and recurrent neural networks were used to distil useful features for classifying abnormalities and diseases. In terms of reasoning, there were two studies based on NLP (24,37) that performed reasoning on a diseasome-patholome KG to form a clinical expert system for expert doctors or unskilled health workers. The KG was used to provide proactive acute care, and to help identify an underlying condition that may or may not be manifesting symptoms.

Several studies have used KGs in machine learning models in in-model XAI systems. Like pre-model XAI, KGs have mainly been used in neural-network-based models for applications related to extraction and reasoning. For example, in Daniels et al. (33) a KG was used to improve a deep learning model’s performance on an image classification problem. To this end, the authors trained the model to predict every node in a knowledge graph and then propagated information between the nodes to refine the predictions. With respect to relation extraction, an RNN algorithm was used (28) to extract the semantic relationships in a text dataset (WordNet^3^) and to find a path between sentences. In Silva et al. (28), the authors used a two-level attention mechanism to capture the hierarchical relationships among medical codes and to transfer knowledge among EHR codes in an effort to overcome extremely imbalanced label distribution. In Riquelme et al. (54), the authors used a residual neural network (ResNet) in a Visual Question Answering (VQA) problem wherein the model supported the answers using image and text explanations. A decision-tree-based model with a KG was also used in Ko et al. (50) to capture the semantic relationships between knowledge elements for the purpose of designing rules in the additive manufacturing domain. With respect to KG reasoning, the authors of Gaur et al. (45) leveraged external knowledge to enable the embedding of KGs in hidden layers in order to generate explainable outcomes by tracing over the KG. Finally, the authors of Yu et al. (24) also used NLP techniques on a tax KG to explain the calculated results and tax refunds through reasoning.

The authors of Ma et al. (49) created a KG (CovidCare) and proposed a transfer-learning-based prognosis solution to predict the length of stay of patients with COVID-19. Regarding post-model XAI, our review found no articles wherein a KG was constructed for explainability purposes after the application of a machine learning model. Instead, we found numerous studies (e.g. Previous works (29,36,39,57)) wherein the KG was applied for reasoning. For example, the authors of Fuji et al. (58) used bioinformatics and medical-literature KGs in conjunction with a deep neural network model to search for explanations via reasoning and to provide corroborating evidence for phenomena wherein only a little is known about the relationships. Likewise, both LSTM and CNN were used in Nikolov and d’Aquin (29) to mine rules in a KG in order to explain the models’ decisions in a text classification problem. In the healthcare domain, the authors of Sun et al. (36) developed rules in a medical KG to assess the clinical rationality of medical claims and to identify the suspected claims by reasoning.

In the reviewed studies, KGs were mostly used for reasoning and inference in post-model XAI, with Deep Learning, CNN, and LSTM being the most commonly used machine learning algorithms. For example, the authors of Sun et al. (36) used a Deep Learning model in conjunction with reasoning via a medical KG to assess the clinical rationality of claims in order to identify the suspected claims. The authors also used a KG to transfer knowledge among Electronic Health Record (EHR) codes in order to reduce extremely imbalanced label distribution. In another study, Futia and Vetrò (47), KGs were used as a backbone for several reasoning mechanisms, ranging from consistency checking to causal inference. Similarly, the authors of Fuji et al. (58) used a KG in the field of bioinformatics to search for knowledge that could provide corroborating evidence for phenomena wherein relationships are only partially known. These reasoning procedures were enabled by ontologies, and they provided a formal representation of the semantic entities and relationships relevant to a specific sphere of knowledge.

Graph neural network models were also prominent in feature extraction or KG construction in post-model XAI systems. As an example, the authors of Xie et al. (52) used a GNN model to capture the hierarchical relationships among medical codes and to transfer knowledge among EHR codes to alleviate extremely imbalanced label distribution.

It should be noted that a few of the reviewed studies used KGs in different XAI systems. For instance, in Fuji et al. (35), It should be noted that a few of the reviewed studies used KGs in different XAI systems. For instance, in Fuji et al. (35), a financial dataset was converted into a KG, which was then applied in conjunction with an inference engine and a deep tensor model. The authors were then able to use their inference engine and financial KG to explain the output and financial results.

## 6. Conclusion

In this article, we have provided a framework for investigating the use of KGs for explainability in XAI and machine learning models. This framework was created by performing a systematic review of recently published studies and examining how and where researchers have used KGs. After reviewing the articles through three specific lenses (explainability, machine learning model, and XAI system type), the following conclusions were derived:

- Many studies have leveraged KGs to extract features, entities, and relations prior to applying an XAI model, as well as for the purpose of inference and reasoning after developing machine learning algorithms.
- KGs have been mainly incorporated in pre-modelling XAI to extract features and relations for different purposes, including extracting entities from text. Several studies also constructed KGs in the pre-modelling phase.
- According to our analysis, KGs have been mostly used for inference and reasoning in the post-model XAI.
- Neural-network-based machine learning models were the most commonly used algorithms for explainability with KGs. Many studies extracted features from text using NLPs, followed by the construction of a KG for explainability purposes.
- Although KGs have been widely utilised in different domains, we found several studies that leveraged KGs to explain the XAI models in healthcare.
